# Application of Dang Van Model Based on Critical Plane Approach for Rolling Contact Problems

**DOI:** 10.3390/ma19102173

**Published:** 2026-05-21

**Authors:** Paweł J. Romanowicz

**Affiliations:** Department of Machine Design and Composite Structures, Faculty of Mechanical Engineering, Cracow University of Technology, ul. Warszawska 24, 31-155 Cracow, Poland; pawel.romanowicz@pk.edu.pl

**Keywords:** rolling contact fatigue, Dang Van criterion, compressive stresses, material properties, multiaxial high-cycle fatigue criteria

## Abstract

Analyses of rolling contact fatigue (RCF) problems require the use of multiaxial fatigue criteria, which take into account complex non-proportional stress conditions. One of the most often used criteria to analyse this phenomenon is the Dang Van criterion. However, this criterion is often criticised due to its overestimation of the influence of compressive stresses on fatigue strength, which leads to an underestimation of the equivalent fatigue stress. Due to the high popularity of this hypothesis, in this paper a few modifications of the Dang Van criterion based on the critical plane approach are compared. One of the investigated modifications is a new proposal in which it is assumed that compressive hydrostatic stresses are as unfavourable as tensile stresses. All variants are verified in three ways: (1) by means of the experimental results for the out-of-phase pulsating compression and alternating torsion; (2) by comparison with the results obtained by means of the Papadopoulos criterion (which provides the most accurate results for RCF issues); and (3) using the example of an RCF analysis of a roller bearing. Based on these investigations, it is confirmed that the original Dang Van criterion is not suitable for application to RCF problems. It is shown that the mere omission of compressive hydrostatic stresses is also insufficient. The highest agreement with the experimental results (relative error δ = 0.77%), the Papadopoulos criterion (δ=5.8%) and, in the case of the practical application (roller bearing; δ=1.1%), is obtained for the proposed modification in which it is assumed that the compressive hydrostatic stress is an unfavourable for fatigue processes in the same way as the tensile hydrostatic stress.

## 1. Introduction

Fatigue of material is one of the most important causes of failure of structures and machine parts [1]. Depending on the number of cycles to failure, this phenomenon can be divided into low-cycle or high-cycle fatigue. Another criterion for differentiation of fatigue is the nature of the stress state. Distinguishing from the simplest to the most general, the load conditions are uniaxial, biaxial and multiaxial. Furthermore, depending on the relationship between the individual stresses, a distinction is made between proportional and non-proportional loads [2].

In the case of simple loads, e.g., uniaxial or proportional, a fatigue analysis can be made using calculation methods based on S-N diagrams and Goodman, Gerber, Smith or Haigh diagrams, etc. [3,4]. In the case of multiaxial stress conditions and, in particular, when non-proportional stresses occur, the use of multiaxial fatigue criteria (MFC) [5] is required. There are a wide range of MFC based on different computational approaches (empirical, critical plane, integral, stress invariants, energy based, etc.) [6,7], but usually only a limited group of criteria are used in multiaxial fatigue analyses. These criteria reduce a complex multiaxial stress state to an equivalent uniaxial stress. They typically require knowledge of two fatigue properties of materials—the alternating fatigue bending (or tension–compression) limit $f_{b-1}$ ${(f}_{tc-1}$) and the alternating torsion fatigue limit $t_{-1}$.

An issue that requires the application of MFC is rolling contact fatigue (RCF). The differences between RCF and classical fatigue are discussed by Sadeghi et al. [8]. Mainly, this phenomenon is characterised by a complex stress state in which three normal zero-based pulsating high-compressive and three fully reversed shear stress components can occur. These stresses are localised in a small volume of material, usually just below the contact surface. Moreover, the shear and normal components are shifted in phase, which results in a non-proportional loading case. When one body rolls over another, this causes the directions of the principal stresses to change their orientation over time at a given point [9].

RCF has been studied in the literature using many different MFC, but a group of the most frequently used criteria can be distinguished. The most commonly used criteria are: the Dang Van (DV) [10,11], the Crossland [12], the Findley [13], the Papadopoulos [14], the Liu and Mahadevan [15], and the Matake [16].

Dang Van proposed two variants of the criterion. The first one [17] is based on the critical plane approach. The second one [10,11], in which the maximum microscopic Tresca’s shear stress is used, is based on a multiscale approach. In both variants, a linear combination of the shear and hydrostatic (which is the first invariant of the stress tensor) stresses is used. The Findley criterion [13] is based on the critical plane approach and takes into account the shear stress amplitude and the maximum normal stress. The Crossland criterion [12] is based on the stress invariant approach. Unlike the DV model, which takes into account the whole distribution of hydrostatic stress over time, the Crossland model only takes into account its maximum value. The Papadopoulos criterion [14] was proposed for hard metals and includes the volumetric root mean square of the resolved shear stress amplitude and the maximal hydrostatic stress. The Liu and Mahadevan criterion [15] belongs to the criteria based on the critical plane deviation. It requires the determination of the fracture material plane in which the highest value of the critical parameter occurs (i.e., the maximum principal stress amplitude). In the next step, the critical plane can be found by rotating the fracture plane by an angle, evaluated based on the material properties [18]. The Matake criterion [16] takes into account the linear combination of the shear stress amplitude and the maximum normal stress. This criterion is frequently cited in the literature and used for RCF analyses.

Both variants of the DV criterion have often been used for fatigue analyses of different machine parts working in RCF loading conditions. In most cases, a criterion based on the second approach, based on the Tresca shear stress, was used. Examples of applications using both criteria are as follows—rails [11], splined parts [19], rolling/sliding contact [20,21,22,23], spherical roller bearings [24,25,26,27], wheel–rail bearings [28], standing contact fatigue [29], railway wheels [30,31], crane wheels [9,25], ollers [32], gears [33], and wind turbine roller bearings [34,35].

Examples of papers that have used the remaining criteria to study RCF issues are as follows:

- The Findley criterion—gears [36], rollers [32], and standing contact fatigue [29];
- The Papadopoulos criterion—spherical roller bearings [24,25], roller bearings [26,27,37], rolling contact fatigue [22,23], and wheels [25];
- The Liu and Mahadevan criterion—spherical roller bearings [24], roller bearings [38], and rolling contact [21];
- The Matake criterion—roller bearings [38], rollers [32], and railway wheels [39,40];
- The Crossland criterion—spherical roller bearings [24,25], railway wheels [25,41], and rolling contact fatigue [23].

In many of the studies that have used the DV criterion to study RCF [9,20,21,22,23,24,25,28,29,34,38], it has been observed that the DV criterion leads to significantly lower fatigue stress than other MFC. This underestimation of the fatigue stress has also been confirmed by comparison with experiments [21,22,29]. This problem is caused by the assumption of the DV criterion that the compressive stress has a positive effect on the fatigue strength. In the case of high compressive stresses, this leads to a significant reduction in the equivalent fatigue stress. Such triaxial compression with high compressive stresses occurs during rolling or standing contact. As a consequence, the level of fatigue effort is underestimated, and the safety factor is overestimated.

Desimone et al. [20] proposed a new conservative limit of the DV criterion. This limit is described by two straight lines. The first one is described by the original DV model. The second one, in which the compressive hydrostatic stress is neglected, is described by a horizontal line whose fatigue limit is reduced from $t_{-1}$ to $0.5f_{-1}$. The disadvantage of this approach is that the condition for the pure alternating torsion test is not fulfilled. This criterion was applied in studies [21,28,34,38] for RCF analyses.

Another modification of the DV criterion was used in Refs. [9,25]. In these studies, the influence of negative (compressive) hydrostatic stress on the fatigue DV stress was neglected. This approach significantly reduced the error obtained for RCF problems, but the fatigue DV stresses were still underestimated.

Despite many critical remarks [9,20,21,22,23,24,28,29,34,38], the Dang Van criterion is still often used in the analysis of structures operating under high compressive stresses [19,31,33]. The high popularity of DV and its widespread use in RCF analyses justifies the need to find a solution (modification) of the DV criterion that guarantees more reliable results for fatigue calculations within RCF issues. In the paper, the first variant of the DV criterion, based on the critical plane approach, is investigated in the context of its application to RCF issues. Despite Desimone’s proposed modification of the DV criterion (for Tresca’s shear stress approach variant), there is no solution for the original version of the DV criterion based on the critical plane. So, the main aim of the study is to propose a new modification of the DV criterion (critical plane approach variant) that is suitable for RCF applications. The existing modifications of the DV criterion, as well as the new proposal, are compared and verified with the use of experimental data. A verification is also performed by a comparison with the results obtained for the Papadopoulos criterion [14], which achieved the highest consistency with the catalogue data for rolling bearings [37]. The Papadopoulos criterion is indicated as suitable for application to RCF problems [22,23]. The analyses also take into account the influence of the criterion calibration method (selection of the material properties) on the calculation results. Finally, a modification of the DV criterion, which significantly more accurately estimates the level of fatigue effort of elements operating under rolling fatigue conditions, is proposed. This is achieved by introducing a modification, namely DV mod. 4, in which it is assumed that compressive stresses unfavourably affect the fatigue strength.

The paper is divided into seven sections. The introduction, literature review and the basis of rolling contact fatigue and methods of RCF analysis are presented in Section 1. The application, comments and modifications regarding the DV criterion are also reported. A detailed description of the DV criterion and its different modifications is given in Section 2. The results are presented and discussed in Section 3. The presented study takes into account the influence of material properties on the results obtained by the use of DV criterion (Section 3.1), an evaluation of the effect of compressive stresses and out-of-phase loading by means of experimental results (Section 3.2), a comparison of results obtained for out-of-phase torsion–compression loading with high compressive stresses (Section 3.3), and a multiaxial fatigue analysis of thrust roller bearings (Section 3.4). A summary and discussion of the obtained results are presented in Section 4. The conclusions are given in Section 5.

## 2. Materials and Methods

### 2.1. Original Dang Van Criterion

The original Dang Van criterion (DV) [11,17,42,43,44] was proposed for fatigue analyses of the structural components subjected to non-proportional multiaxial stress states. The motivation to create this model was its potential application to rolling contact fatigue problems.

RCF is characterised by a specific multiaxial stress state, which consists of high three-dimensional compression and fully reversed torsion with a shift in phase. In such a case, the application of typical simple methods (such as the Gough–Pollard model [45], which is suitable only for proportional loading conditions) for the calculation of the fatigue effort is not possible.

The DV criterion belongs to the group of MFC based on the so-called critical plane approach. It is assumed that material cracking is initiated within the material grain and related to the easiest slip plane. The equivalent DV fatigue stress $\tau_{DV}$ is calculated as the sum of the shear stress τ and the hydrostatic stress $\sigma_{H}$ acting on the critical plane according to the formula

(1) $$\tau_{DV}\left(t\right)=\tau \left(t\right)+a_{DV}\cdot \sigma_{H}\left(t\right)\leq t_{-1},$$

where $t_{-1}$ is the alternating torsion fatigue limit.

The hydrostatic stress $\sigma_{H}$, which is also the first stress invariant, can be calculated as follows:

(2) $$\sigma_{H}=\frac{1}{3}\left(\sigma_{1}\left(t\right)+\sigma_{2}\left(t\right)+\sigma_{3}\left(t\right)\right),$$

where $\sigma_{1},\;\sigma_{2},\;and\;\sigma_{3}$ are the principal stresses, arranged from the highest to the lowest stress.

**Figure 1:**
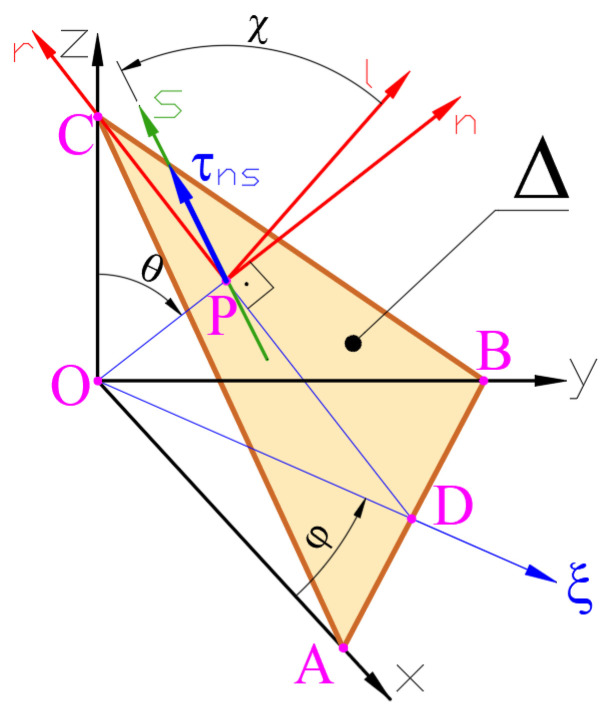
Material plane ∆ (defined by angles φ and θ) passing through point O, where point P tends to point O. Direction of shear stress vector $\tau_{ns}$ in plane ∆ is defined by angle χ.

In order to determine the critical plane during the considered time $\left(t\right)$, all possible material planes should be considered, and the one in which the $\tau_{DV}$ stress reaches its maximum value must be found. Due to the fact that $\sigma_{H}$ is the stress invariant (see Equation (2)), the location of the critical plane is determined by a scalar value defining the length of the vector of the shear stress τ on the searched planes (see Figure 1).

According to the inequality in (1), fatigue failure will occur if the value of the equivalent DV stress $\tau_{DV}$ exceeds the fatigue limit $t_{-1}$. For the determined critical plane, the criterion (1) takes the following form:

(3) $$\tau_{DV}^{MAX}=\underset{V,t}{\max}\left\{\tau_{DV}\right\}\leq t_{-1},$$

where V is the volume of an investigated structure and t is time.

The criterion requires the values of two fatigue material properties—the uniaxial tension–compression $f_{-1}=f_{tc-1}$ [10,22,30] or the alternating fatigue bending limit $f_{-1}=f_{b-1}$ [11] and the alternating torsion fatigue limit $t_{-1}$. In the original formulation [10], the DV constant $a_{DV}$ is defined as follows:

(4) $$a_{DV}=\frac{t_{-1}-\left(f_{-1}/2\right)}{f_{-1}/3}.$$

and for $f_{-1},$ the tension–compression fatigue limit, $f_{tc-1}$ is used.

The above dependency can be written in the following form, which will be used in the further part of this work:

(5) $$a_{DV}=\frac{3t_{-1}}{f_{-1}}-1.5\;.$$

**Figure 2:**
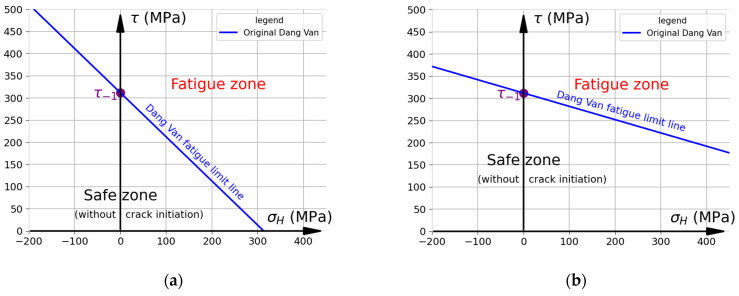
Representation of original Dang Van criterion for R7T steel. Fatigue limits: (**a**) $f_{-1}=f_{tc-1}=375$ MPa and $t_{-1}=312\;$ MPa; (**b**) $f_{-1}=f_{b-1}=520$ MPa and $t_{-1}=312\;$ MPa.

A graphical representation of the original DV criterion is shown in Figure 2. The fatigue limit line (drawn in blue, when $\tau_{DV}=t_{-1}$) separates the safe area (in which cracks should not initiate) from the zone where crack initiation and fatigue failure are assumed to occur. It is assumed that fatigue failure will occur if any point of load history (even just one) plotted in the τ (the shear stress at the critical plane) and $\sigma_{H}$ coordinates exceeds the critical fatigue limit line. Obviously, the original DV fatigue limit line intersects the vertical axis at a point with a value $t_{-1}$. The DV criterion is vulnerable to the material properties (namely, $f_{tc-1}$ or $f_{b-1}$) used for the calibration. This mainly concerns the selection of the fatigue limit for normal stresses. There are two approaches used in the literature (in which $f_{-1}=f_{tc-1}$—Figure 2a; or $f_{-1}=f_{b-1}$—Figure 2b), which significantly complicates the selection of the criterion calibration method. The diagrams were drawn using R7T steel as an example. The fatigue properties ($f_{tc-1}=375$ MPa; $f_{b-1}=520$ MPa; $t_{-1}=312$ MPa) used to draw the fatigue limit lines are based on the data reported in Ref. [22]. The chemical composition of R7T steel is as follows: C—0.49; Mn—0.75; Si—0.35; Cr—0.15; Ni—0.05; Mo—0.02; Cu—0.10; S—0.005; P—0.10; and Fe—rem (in weight%) [22]. The remaining typical mechanical properties of this steel are as follows: the ultimate tensile strength: 820–940 MPa; the Yield limit: 520 MPa; the Young modulus: 210 GPa; and Poisson’s coefficient: 0.3 [46]. It should be noted that the calibration method significantly affects the obtained results of fatigue analyses. A comparison of the influence of the material properties on the fatigue limit lines is presented for each investigated criterion. Generally, using $f_{tc-1}$ as the fatigue limit (instead of $f_{b-1})$ increases the slope of the fatigue limit line. Consequently, this results (Figure 2a) in:

(a) The DV criterion predicting failure at lower tensile hydrostatic stresses $\sigma_{H}$;
(b) The compressive hydrostatic stress $\sigma_{H}$ having a significantly greater positive effect on fatigue life than when using $f_{b-1}$ as the material properties (Figure 2b).

#### Methods for Determining Shear Stresses $\tau \left(t\right)$

In the original DV criterion, it was proposed to use the time-varying shear stress distribution $\tau_{ns}\left(t\right)$ defined in the material plane Δ and in the direction defined by the angle χ:

(6) $$\tau_{ns}\left(t\right)=\tau \left(\varphi ,\theta ,\chi ,t\right)\left|\begin{array}{c} \Delta =\Delta \left(\varphi ,\theta \right)\;,\chi =\mathrm{const} \end{array}\right..$$

Due to the fact that the average shear stress $\tau_{ns,m}$ does not affect the fatigue strength, the amplitude function of stress $\tau_{a,T}(t)$ is used in Equation (1) as the shear stress τ:

(7) $$\tau_{a,T}(t)=\left|\tau_{ns}(t)-\tau_{ns,m}\right|.$$

There are several methods for the calculation of the mean value of stress in a fixed material plane Δ, such as the minimum circumscribed circle, the longest projection and the longest chord [47]. In simple cases, the following formula can be used:

(8) $$\tau_{ns,m}=\frac{\underset{t}{\max}\left\{\tau_{ns}(t)\right\}+\underset{t}{\min}\left\{\tau_{ns}(t)\right\}}{2}.$$

In the second variant of the DV criterion [10], named here as DV_TG_, the Tresca–Guest $\tau_{TG}(t)$ shear stress is used in Equation (1) instead of the shear stress $\tau \left(t\right)$. This $\tau_{TG}$ stress is always positive and does not depend on the orientation of the material plane:

(9) $${\tau \left(t\right)=\tau }_{TG}\left(t\right)=\frac{\sigma_{1}\left(t\right)-\sigma_{3}\left(t\right)}{2},$$

where $\sigma_{1},\;\sigma_{2},\;and\;\sigma_{3}$ are the principal stresses.

Since the Tresca–Guest $\tau_{TG}(t)$ shear stress is used as the shear stress $\tau \left(t\right)$, the direction of the ${\tau \left(t\right)=\tau }_{TG}\left(t\right)$ stress vector may change over time and may occur in different material planes and directions χ. So, the critical plane approach is not used in this approach. Because of this, the DV_TG_ approach is not consistent with the original DV model.

### 2.2. Modifications and Improvements of Dang Van Criterion

In the original Dang Van criterion, it is assumed that the compressive stresses have a positive effect on the fatigue strength, which means that the greater the compression, the greater the shear stress that must occur to initiate cracks and material fatigue. However, based on verifications through the experiments, it has been reported in many papers that the DV criterion overestimates the influence of this effect. This particularly applies to the RCF phenomenon, in which very high three-directional compression occurs. In such cases, it has been observed that the compressive stresses, instead of having a positive effect on the fatigue strength, result in a reduction in the fatigue resistance. Due to this fact, some modifications of the Dang Van criterion have been proposed in the literature.

#### 2.2.1. Correction of $a_{DV}$ for Materials with Fatigue Limits t_−1_/f_−1_ < 0.5 (DV mod. 1)

The parameter $a_{DV}$ (Equation (5)) depends on two fatigue limits, $t_{-1}$ and $f_{-1}$, of the investigated material and takes into account the influence of the hydrostatic stresses on the fatigue process. A positive value of $a_{DV}$ results in the tensile stress lowering the allowable shear stress $\tau \left(t\right)$, which is consistent with physical observations. This rule is fulfilled for materials in which $t_{-1}>0.5f_{-1}$. If $t_{-1}=0.5f_{-1}$, then $a_{DV}$= 0, and the DV criterion does not take into account the influence of either the compressive or tensile stresses on the fatigue strength. In the case of materials for which $t_{-1}<0.5f_{-1}$, the parameter $a_{DV}$ is negative and means that the tensile stresses have a positive effect on material fatigue, which is not physically possible. In order to avoid this problem, the following modification of the DV criterion (this change concerns the dependencies described by Equations (4) and (5)) was proposed [26]:

(10) $$a_{DV}=\left\{\begin{array}{c} 0\;\;\;\;\;\;\;\;\;\;\;\;\;\;\;\;\;\;\;\;\;\;\;\;if\;\;\;\frac{t_{-1}}{f_{-1}}\leq 0.5\\ \left(\frac{3t_{-1}}{f_{-1}}-1.5\right)\;if\;\;\frac{t_{-1}}{f_{-1}}>0.5 \end{array}\right.\;.$$

Because the tested materials in this study are characterised by the dependence $\frac{t_{-1}}{f_{-1}}>0.5$, this modification is not investigated.

#### 2.2.2. Modification Proposed by Desimone et al. (DV mod. 2)

**Figure 3:**
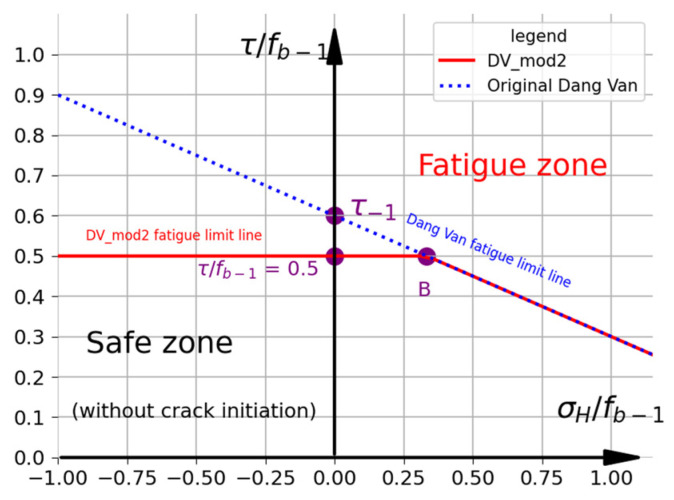
Representation of DV mod. 2 criterion (R7T steel). Fatigue limits: $f_{-1}=f_{b-1}=520$ MPa and $t_{-1}=312\;$ MPa.

Based on the observations of the stress histories for bending and axial experiments carried out under different stress ratios, Desimone et al. [20] proposed a conservative modification of the DV criterion for RCF applications. The fatigue limit line (Figure 3) is divided into two parts: horizontal, in which $a_{DV}=0$, and inclined, in which $a_{DV}$ is described by the general DV formula (Equations (4) and (5)). Based on the Tresca–Guest hypothesis (Equation (9)), point B determines the position of the maximum tensile stress for the alternating fatigue bending test ($\tau =0.5f_{b-1};\;\sigma_{H}=f_{b-1}/3$). On this basis, in this approach, the fatigue limit for the alternating torsion is limited to only $0.5f_{b-1}$. The assumed limit is generally significantly smaller than the alternating torsion fatigue limit $t_{-1}$. It should be noted that in study [20], the authors calculated $\tau \left(t\right)$ with the use of the Tresca–Guest shear stress (see Equation (9)). Therefore, for a small $\sigma_{H}$, this approach is very conservative and the condition for the alternating torsion fatigue limit is not met. In summary, the modification presented in Figure 3 can be described as follows:

(11) $$a_{DV}=\left\{\begin{array}{c} 0\;\;\;\;\;\;\;\;\;\;\;\;\;\;\;\;\;\;\;\;\;\;\;\;if\;\;\;\sigma_{H}<\frac{f_{b-1}}{3}\\ \left(\frac{3t_{-1}}{f_{-1}}-1.5\right)\;if\;\;\sigma_{H}\geq \frac{f_{b-1}}{3} \end{array}\right.\;,$$

and

(12) $$\;\tau_{DV}^{MAX}=\underset{V,t}{\max}\left\{\begin{array}{c} \tau \left(t\right)+a_{DV}\cdot \sigma_{H}\left(t\right)\leq 0.5f_{b-1}\;\;\;\mathrm{or}\;\sigma_{H}<\frac{f_{b-1}}{3}\\ \tau \left(t\right)+a_{DV}\cdot \sigma_{H}\left(t\right)\leq t_{-1}\;\;\;\mathrm{or}\;\sigma_{H}\geq \frac{f_{b-1}}{3} \end{array}\right..$$

#### 2.2.3. Modification in Which Compressive Hydrostatic Stress Is Neglected (DV mod. 3)

In this variant, the compressive (negative) hydrostatic stresses $\sigma_{H}$ are neglected in the DV criterion [9,25]. For the tensile (positive) stresses, the original DV formulation is used. The criterion is described by the following formulations:

(13) $$\tau_{DV}(t)=\left\{\begin{array}{c} \tau \left(t\right)+a_{DV}\cdot \sigma_{H}\left(t\right)\;\;\;if\;\;\;\sigma_{H}\geq 0\;\\ \tau \left(t\right)\;\;\;\;\;\;\;\;\;\;\;\;\;\;\;\;\;\;\;\;\;\;\;\;\;\;\;\;\;if\;\;\;\sigma_{H}<0 \end{array}\right.\leq t_{-1}.$$

**Figure 4:**
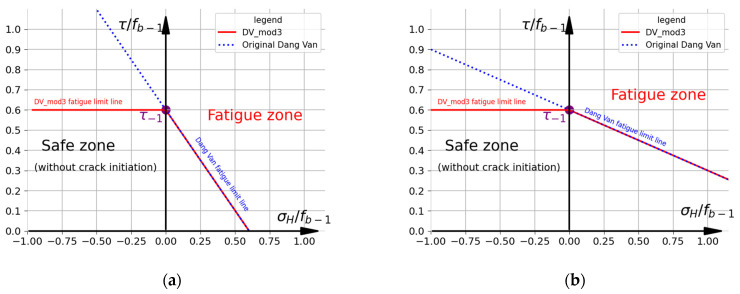
Representation of DV mod. 3 criterion (R7T steel). Fatigue limits: (**a**) $f_{-1}=f_{tc-1}=375$ MPa and $t_{-1}=312\;$ MPa; (**b**) $f_{-1}=f_{b-1}=520$ MPa and $t_{-1}=312\;$ MPa.

The graphical representation of this approach is shown in Figure 4.

#### 2.2.4. Modification in Which It Is Assumed That Compressive Stress Is Unfavourable (DV mod. 4)

Based on the previous author’s studies [9,25,37] and the results of the out-of-phase experimental fatigue tests with alternating torsion and pulsating compression [22], it is assumed that compressive hydrostatic stresses have a negative influence on the fatigue resistance. Consequently, both tensile and compressive hydrostatic stresses reduce the fatigue strength under multiaxial loading conditions with shear stresses. For this purpose, the additional compressive stress influence constant $a_{c}$ is introduced. This constant determines the effect of compressive stresses on material fatigue in relation to the effect of tensile stresses. Finally, the DV mod. 4 is described by the following relationship:

(14) $$\tau_{DV}(t)=\left\{\begin{array}{c} \tau \left(t\right)+a_{DV}\cdot \sigma_{H}\left(t\right)\;\;\;\;\;\;\;\;\;\;\;\;\;\;\;\;\;\;\;if\;\;\;\;\;\;\;\;\;\;\sigma_{H}\geq 0\;\\ \tau \left(t\right)\;+a_{DV}\cdot a_{c}\cdot \left|\sigma_{H}\left(t\right)\right|\;\;\;\;\;\;\;\;\;\;\;if\;\;\;\;\;\;\;\;\;\;\sigma_{H}<0 \end{array}\right.\leq t_{-1}.$$

The constant $a_{c}$ modifies the inclination angle of the fatigue limit line on the side of the compressive hydrostatic stresses with respect to the inclination angle on the side of the tensile hydrostatic stresses. The following cases can be distinguished:

- $a_{c}<0$—The compressive hydrostatic stress has a positive influence on the fatigue strength.
- $a_{c}=-1$—DV mod. 4 corresponds to the original DV criterion.
- $a_{c}=0$—The hydrostatic stresses are omitted in the analyses. DV mod. 4 corresponds to DV mod. 3.
- $a_{c}>0$—The compressive hydrostatic stress has a negative influence on the fatigue strength.
- $a_{c}=1$—The compressive stresses reduce the fatigue strength to the same extent as the tensile stresses.

**Figure 5:**
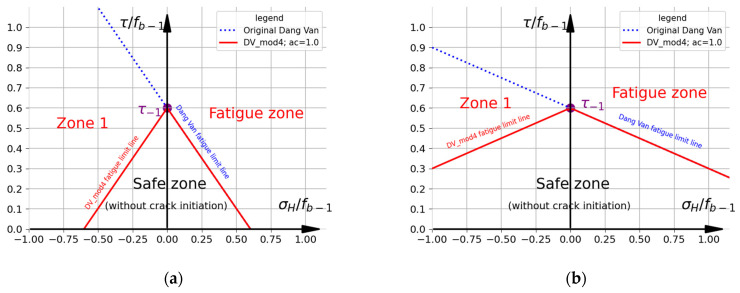
Representation of DV mod. 4 criterion (R7T steel; $a_{c}=1$). Fatigue limits: (**a**) $f_{-1}=f_{tc-1}=375$ MPa and $t_{-1}=312\;$ MPa; (**b**) $f_{-1}=f_{b-1}=520$ MPa and $t_{-1}=312\;$ MPa.

In the case of non-proportional pulsating compression with alternating torsion, fatigue cracks are caused by shear stresses and deformations. However, the presence of compressive stresses changes the distribution of stresses and the principal stress directions. It is also observed that in such a case, the large amount of compressive stress could dominate secondary crack formation [48]. These effects may activate additional slip systems, which could consequently accelerate fatigue degradation and reduce fatigue life. Because of this, in this study, the initial value $a_{c}=1$ is assumed. This value is based on the shape of classical Smith’s chart and the relationship between the tensile and the compressive strength of steel, for both static and fatigue conditions. Additional calculations are made for the coefficients $a_{c}=0.8$ and $a_{c}=0.5$. The graphical representation of this criterion is shown in Figure 5.

#### 2.2.5. Calculation Procedure Using DV Criterion Based on the Critical Plane Approach

The following procedure is used for the determination of the maximal equivalent Dang Van fatigue stress ($\tau_{DV}^{MAX}$):

1. Determination of variations in the time of the stress tensor at the investigated points.
2. Calculation of $\tau_{DV}^{MAX(P_{i})},$ which is the maximal value of $\tau_{DV}(t)$ at the point $P_{i}$. Determination of the orientation of the material plane ${∆}_{cr}^{P_{i}}\;=\;∆\left(\varphi ,\theta \right)$ and angle χ at which $\tau_{DV}^{MAX(P_{i})}$ achieves its maximal value. Here, the angle χ specifies the direction of the shear stress vector $\tau_{ns}$ in the plane ∆, and ${∆}_{cr}^{P_{i}}$ is the critical plane of the point $P_{i}$:
  (a) Calculation of the shear stress $\tau_{ns}\left(t\right)$ at the point $P_{i}$, in the direction of the vector $\vec{s}$ (defined by the angle χ) on the considered material plane ∆ (defined by angles φ and θ);
  (b) Computation of the mean value of shear stress $\left(\tau_{ns,m}\right)$ at the point $P_{i}$ in the plane ∆ and in the direction $\vec{s}$ defined by the angle χ;
  (c) Calculation of the amplitude function of stress $\tau_{a,T}(t)$ in the plane ∆ and in the direction $\vec{s}$ over time t;
  (d) Determination of the hydrostatic stress $\sigma_{H}(t)$ at the point $P_{i}$ over time t;
  (e) Computation of the DV fatigue stress $\tau_{DV}\left(t\right)$ for a certain material plane ∆ and direction $\vec{s}$ at the point $P_{i}$. Comparison with values of $\tau_{DV}\left(t\right)$ obtained for the other remaining configurations of ∆ and $\vec{s}$ at the point $P_{i}$. The maximal value of $\tau_{DV}\left(t\right)$ and its orientation (∆ and $\vec{s}$) is sought.

The calculation step (determining the orientations of material planes ∆) is assumed to equal 5 degrees for each angle φ, θ and χ.

3. Calculation and comparison of the maximal values of $\tau_{DV}^{MAX(P_{i})}$ obtained for the other points $P_{i}$ in the investigated area. The maximal value $\tau_{DV}^{MAX}=\underset{V}{\max}\left\{\tau_{DV}^{MAX\left(P_{i}\right)}\right\}$ is considered to be the equivalent DV fatigue stress value and its orientation (∆ and $\vec{s}$) is called the critical plane.

### 2.3. Papadopoulos Hypothesis

The Papadopoulos criterion [14] was proposed for hard metals. Its main assumption is that the phase difference in out-of-phase bending and torsion does not affect the fatigue strength. The main parameter of this criterion is the volumetric root mean square of the resolved shear stress amplitude $\langle T_{a}\rangle$. The second major difference from the DV is that the Papadopoulos criterion takes into account only the maximal (for the entire period in time) value of the hydrostatic stress $\sigma_{H,MAX}$. The equivalent fatigue stress (with respect to the material plane defined in Figure 1) can be calculated as

(15) $$\tau_{P1,MAX}=\langle T_{a}\rangle +a_{P1}\cdot \sigma_{H,MAX}\leq t_{-1},$$

where

(16) $$\langle T_{a}\rangle =\sqrt{\frac{5}{8\pi^{2}}\int_{\varphi =0}^{2\pi }\int_{\theta =0}^{\pi }\int_{\chi =0}^{2\pi }\tau_{a}^{2}\left(\varphi ,\theta ,\chi \right)d\chi \cdot \sin\left(\theta \right)d\theta \;d\varphi }\;,$$

(17) $$a_{P1}=\frac{3t_{-1}}{f_{-1}}-\sqrt{3},$$

(18) $$\tau_{a}\left(\phi ,\theta ,\chi \right)=0.5\left[\underset{t\in T}{max}\;\tau \left(\varphi ,\theta ,\chi ,t\right)-\underset{t\in T}{min\;}\tau \left(\varphi ,\theta ,\chi ,t\right)\right].$$

The Papadopoulos criterion has been indicated as the criterion that can predict the right relationship between the allowable shear stress amplitude and the out-of-phase negative normal stresses [22]. The research performed by Ciavarella et al. [23] also indicated the Papadopoulos criterion as suitable for rolling contact fatigue applications. It has also been successfully applied to different analyses of RCF problems [9,37]. In the case of larger-sized structures (e.g., rollers with a diameter greater than 8 mm), the additional introduction of the size factor ($k_{s}\leq 1$)

(19) $$\tau_{P1,MAX}=\frac{1}{k_{s}}\langle T_{a}\rangle +a_{P1}\cdot \sigma_{H,MAX}\leq t_{-1}$$

allowed for the achievement of high compatibility with the catalogue data [37]. Due to its best fit to the catalogue data for the parts operating in rolling contact conditions, the Papadopoulos criterion is adopted in this work as the reference criterion for verifying the DV criteria.

## 3. Results

### 3.1. Effect of Material Properties

**Figure 6:**
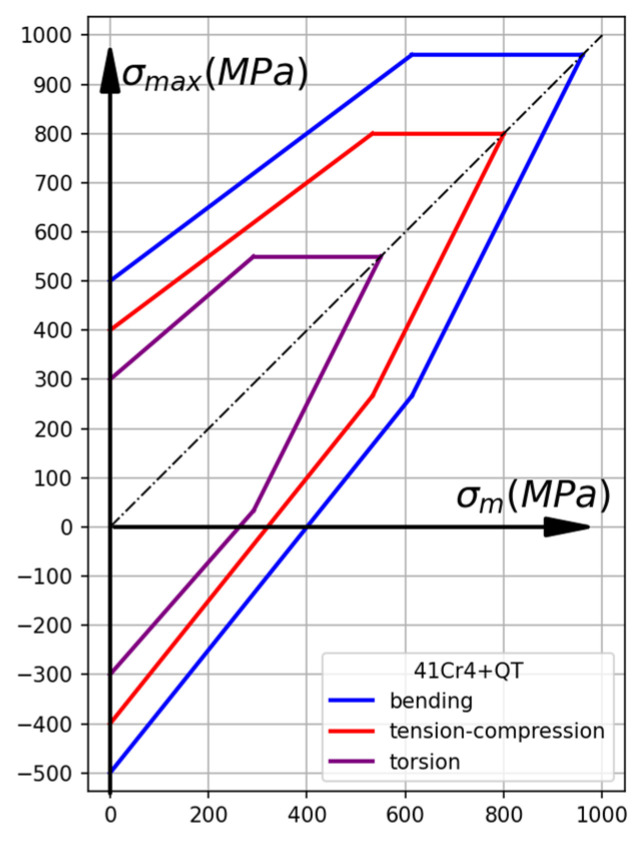
Smith’s chart for 41Cr4 + QT steel.

The fatigue strength of steels depends on many factors, but the most important are the type of load and the stress ratio. When sorting from the highest to the lowest fatigue strength, the following order is observed: bending > tension–compression > torsion. This relationship is illustrated in the Smith chart (Figure 6) for the example of 41Cr4+QT steel [49]. Generally, the fatigue strength for the tension–compression $f_{tc-1}\;$ is equal to about 0.7–0.8 of the alternating fatigue bending limit $f_{b-1}$.

In the case of the DV criterion, the coefficient determining the influence of hydrostatic stresses on the equivalent fatigue effort can be calculated based on $f_{tc-1}$ or $f_{b-1}$. Based on the fatigue data reported in the literature for different common steels [22,49], the Dang Van coefficients $a_{DV}$ were calculated and are reported in Table 1. These calculations were made using the uniaxial tension–compression fatigue limit $f_{tc-1}$ ${(a}_{DV(t-c)})$ and the alternating fatigue bending limit $f_{b-1}$ ${(a}_{DV(b)})$. It can be seen that $a_{DV}$ mainly depends on the type of fatigue data used. When $f_{tc-1}$ was used, then $a_{DV}=a_{DV(t-c)}$ reached values above 0.7. When $f_{b-1}$ was used, then $a_{DV}=a_{DV(b)}$ was in a range of about 0.25–0.35. A higher value of $a_{DV}$ results in the stronger influence of hydrostatic stress on the equivalent fatigue effort. In the original DV, a positive tensile hydrostatic stress increases the equivalent fatigue effort, but a negative compressive hydrostatic stress decreases the equivalent fatigue effort. This is particularly important in the case of RCF, in which the hydrostatic stress reaches large negative values (high three-dimensional compression). In such a situation, when the $a_{DV}$ coefficient is large, it leads to a situation in which the compressive (negative) stress strongly reduces the influence of shear effects. This may result in a major underestimation of the fatigue effort and a potential fatigue failure.

The influence of the selection of material properties on the results obtained by the original DV criterion is illustrated in Table 2. Here, the calculations are performed for 30CrNiMo8 + QT steel for out-of-phase loading conditions (alternating torsion with pulsating tension or compression; shift in phase, 90°). The principal mechanical properties of the steel were as follows: the ultimate tensile strength: 1250 MPa; the Yield limit: 900–1050 MPa; the Young modulus: 217 GPa; and Poisson’s coefficient: 0.3. The chemical composition can be found in Ref. [9]. The analyses include the use of two $a_{DV}$ coefficients calculated on the basis of fatigue limits for the tension–compression $f_{tc-1}$ and the alternating bending $f_{b-1}$. In the case of tensile stresses, a higher fatigue effort is obtained when the fatigue limit for tension–compression ($a_{DV(t-c)}=0.75$) is used. On the other hand, this configuration of material properties results in a lower fatigue effort (in comparison with the case with $a_{DV(b)}=0.30$ calculated based on $f_{b-1}$) when there is a state of compressive stress. However, it should be noted that in both investigated cases, the compressive stresses significantly reduce the equivalent fatigue effort. This effect has been criticised in the literature [9,20,21,22,23,24,25,28,29,34,38] because it leads to underestimation of the fatigue stress.

### 3.2. Evaluation of Effect of Compressive Stresses and Out-of-Phase Loading by Means of Experimental Results

The validation of the original DV criterion, as well as its modifications, was compared using two examples. In the first case, the results of the non-proportional biaxial fatigue experiments published by Bernasconi et al. [22] were used. These tests were performed for R7T wheel steel grade (with the chemical composition and material properties reported in Section 2.1), which is commonly used in railway applications. The samples were loaded by pulsating compression (with stress amplitude $\sigma_{a}$ and mean value $\sigma_{m}$) and alternating torsion (with amplitude $\tau_{a}$), with a shift in phase equal to 90°. The values of the allowable shear stress amplitude $\tau_{a}$ were determined using a staircase test sequence, with an increment of 15 MPa and an assumed fatigue life of $3\cdot 10^{6}\;cycles$. More details, including the information about the Standard Deviation and the number of specimens, can be found in [22]. The key test results used in this validation are reported in Table 3. The fatigue limits obtained for this steel were as follows: $f_{tc-1}=375$ MPa (the tension–compression) and $t_{-1}=312\;$ MPa (the fully reversed torsion) [22]. The fatigue limit for fully reversed bending was estimated based on the typical relationship for steel, $t_{-1}=0.6f_{b-1}$. According to this relationship, for example, for $t_{-1}=312\;MPa$, the sought value of the fatigue limit for fully reversed bending is $f_{b-1}=520\;MPa$. The specimens were cut from the wheel in two different directions (axial 1–3; circumferential 4–6); hence, the results show little anisotropy.

In order to compare and evaluate the different variants of the DV and the Papadopoulos criteria, the following relative error values (δ) were used:

(20) $$\delta =\frac{\tau_{DV}^{MAX}-\tau_{LIM}}{\tau_{LIM}}100\%,\;\;\;\;\delta =\frac{\tau_{P1,MAX}-\tau_{LIM}}{\tau_{LIM}}100\%,$$

where $\tau_{LIM}=t_{-1}$ or $\tau_{LIM}=0.5f_{b-1}$, depending on the criterion used.

Additionally, for the investigated cases, the following mean error values were calculated—the simple arithmetic mean $\delta_{mean}$ and the arithmetic mean of absolute values $\delta_{m\_abs}$:

(21) $$\delta_{mean}=\frac{\sum_{i=1}^{n}\delta }{n},\;\;\;\;\;\;\;\delta_{m\_abs}=\frac{\sum_{i=1}^{n}\left|\delta \right|}{n}.$$

The simple arithmetic mean $\delta_{mean}$ shows the overall trend of whether the criterion is conservative or not conservative. The second one, $\delta_{m\_abs},$ shows the average error value calculated from the absolute values.

The validation of the DV criteria was made for three cases. In the first case, the coefficient $a_{DV}=0.996$ was calculated on the basis of $f_{tc-1}=375$ MPa (in the same way as in Ref. [22]). In the second case, coefficient $a_{DV}$ was calculated with the use of $f_{b-1}=520$ MPa, and $a_{DV}=0.3$. In both cases, anisotropy was not taken into account when determining the $a_{DV}$ and fatigue limit $\tau_{LIM}$ values. In the third case, the anisotropy of the tested material (samples were cut from the wheel rig) was included in the analyses. This was made by taking into account the changes in the material properties to calculate the $a_{DV}$ and the fatigue limit $\tau_{LIM}$. The $a_{DV}$ (Equations (10) or (11)) was calculated using the fatigue limit for the alternating fatigue bending limit, $f_{b-1}=t_{-1}/0.6$. Finally, the following fatigue limits were used—$\left(t_{-1}=297\;MPa;\;f_{b-1}=495\;MPa\right)$ and $\left(t_{-1}=310\;MPa;\;f_{b-1}=517\;MPa\right)$ for tests No. 1–No. 3 and tests No. 4–No. 6, respectively. The fatigue limits $t_{-1}$ were taken from Ref. [22].

Additionally, the calculations were carried out with the use of the Papadopoulos criterion [14], which has been indicated in the literature [22,23] as one of the most accurate MFC describing the fatigue effort of RCF.

**Figure 7:**
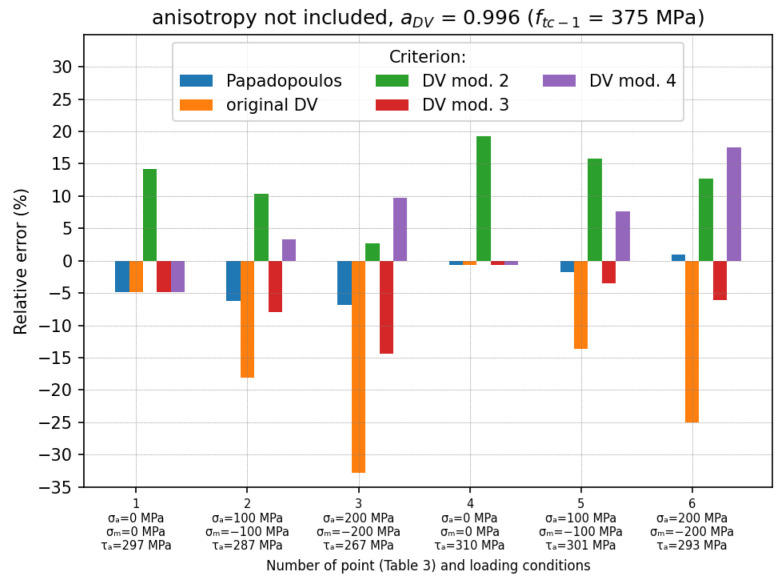
Relative errors for loading cases described in Table 3; anisotropy of material is not included and $a_{DV}=0.996$ ($f_{tc-1}=375$ MPa is used).

The results of the calculations are presented in the form of relative error (see Equation (20)) for each of the tested points (see Table 3). In the first case (Figure 7), in which $f_{tc-1}=375$ MPa was used for the determination of $a_{DV}$, the use of the original DV criterion led to a significant underestimation of the fatigue effort (mean error $\delta_{mean}=-15.83\%$). This was due to the high value of the $a_{DV}=0.996$ coefficient, which caused the negative hydrostatic stresses to be subtracted from the shear stress τ, and consequently reduced the level of $\tau_{DV}$ stress. It can be seen that the underestimation of $\tau_{DV}$ stress increased with an increase in the negative $\sigma_{H}$. This effect of $\sigma_{H}$ stress was reduced when the DV mod. 4 was used ($\delta_{mean}=5.46\%,\;\delta_{m\_abs}=7.28\%$). However, in this case, the error also increased with an increasing compressive $\sigma_{H}$ (but in this case, there was an overestimation of $\tau_{DV}$).

**Figure 8:**
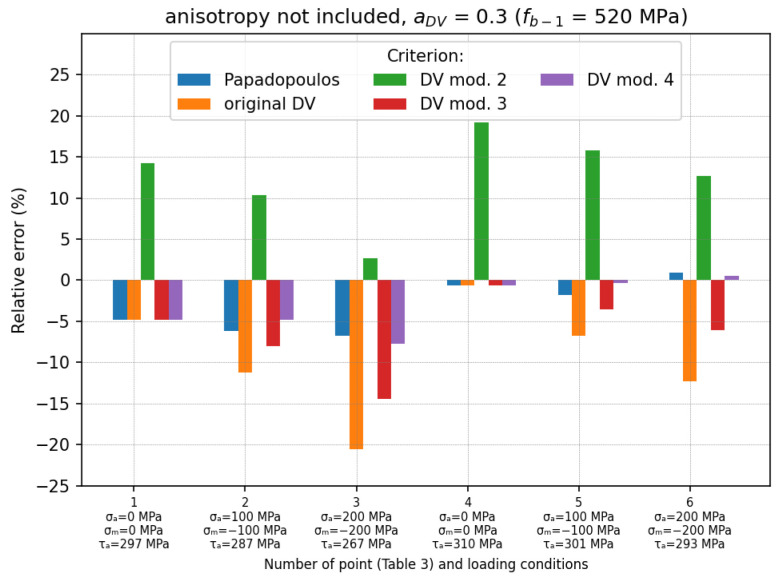
Relative errors for loading cases described in Table 3; anisotropy of material is not included and $a_{DV}=0.3$ ($f_{b-1}=520$ MPa is used).

The remaining criteria (the Papadopoulos, the DV mod. 2 and the DV mod. 3) did not take into account the influence of negative $\sigma_{H}$ stress. Because of this, the value $a_{DV}$ had no effect on the results of $\tau_{DV}$ calculations. It can be seen that these results (Figure 7—obtained by these three criteria) were the same as for the second case (Figure 8), regardless of $a_{DV}$. The smallest mean error was obtained for the Papadopoulos criterion (underestimation: $\delta_{mean}=-3.21\%,\;\delta_{m\_abs}=3.52\%$). The DV mod. 3 also underestimated the $\tau_{DV}$ stress, with the mean error $\delta_{mean}=-6.25\%$. On the other hand, DV mod. 2 overestimated the $\tau_{DV}$ stress due to the lower fatigue limit ($\tau_{LIM}=0.5,\;f_{b-1}=260\;MPa$).

The results obtained for the second case ($a_{DV}=0.3$ calculated using $f_{b-1}=520$ MPa; anisotropy omitted) are shown in Figure 8. As mentioned above, the results obtained for the Papadopoulos, the DV mod. 2 and the DV mod. 3 criteria were the same as for the first case and will not be discussed again here. However, it should be noted that when $f_{b-1}$ was used, the $a_{DV}$ coefficient was significantly smaller (about three times—see Table 1) compared to the case in which $f_{tc-1}$ was used. This resulted in the much smaller impact of the hydrostatic stresses on the fatigue effort. For example, the mean errors obtained by the original DV and the DV mod. 4 criteria were reduced to $\delta_{mean}=-9.38\%$ and $\delta_{mean}=-2.97\%\;(\delta_{m\_abs}=3.15\%)$, respectively. It can be seen that in this case, the results obtained using DV mod. 4 were comparable to those obtained with the use of the Papadopoulos criterion.

**Figure 9:**
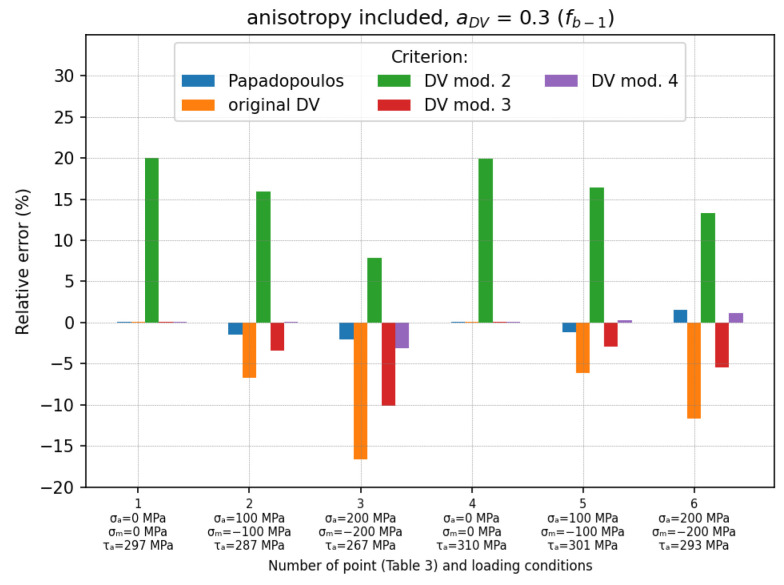
Relative errors for loading cases described in Table 3; anisotropy of material is included and $a_{DV}=0.3$ ($f_{b-1}$ is used).

The third case (Figure 9) differed from the second one (Figure 8) in that the anisotropy of the tested samples was additionally taken into account during the calculation. This was done by using the appropriate material values ($f_{b-1}$ and $t_{-1}$). In this case, except for the DV mod. 2, all the criteria provided smaller mean errors than in the previous cases. The smallest mean error was obtained for the DV mod. 4 ($\delta_{mean}=-0.27\%$ and $\delta_{m\_abs}=0.77\%$). The larger error (very conservative) for the DV mod. 2 was caused by an arbitrarily assumed lower fatigue limit than the true one ($\tau_{LIM}=0.5f_{b-1}$).

The relative errors for each loading condition are shown in Figure 7, Figure 8 and Figure 9. The final summary with the mean relative errors is given in Table 4.

### 3.3. Comparison of Results Obtained for Out-of-Phase Torsion–Compression Loading with High Compressive Stresses

**Figure 10:**
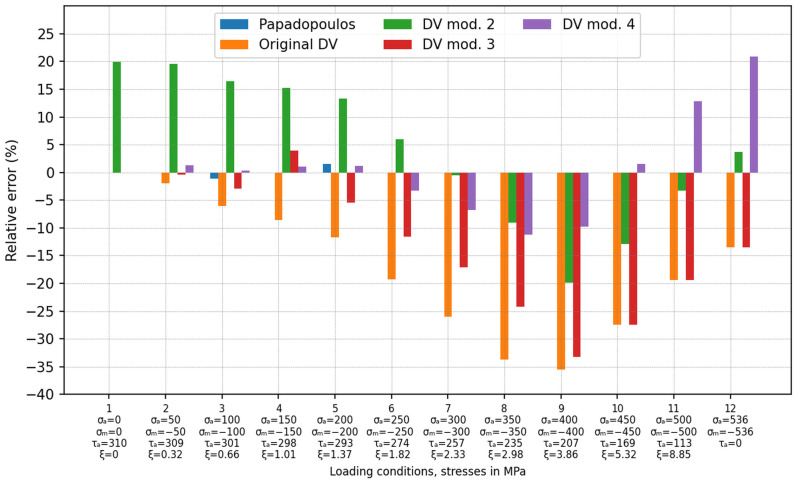
Relative errors for loading cases with large compressive stresses: steel R7T, $a_{DV}=0.3$ ($t_{-1}=310\;MPa;\;f_{b-1}=517\;MPa$).

The validation of the DV criteria presented in the previous subsection was based on experimental results performed using a limited compressive stress range. Based on the data presented in Figure 9, it can be seen that the value of the compressive stress significantly affected the relative error level. Therefore, further verification of the presented modifications of the DV criterion for higher compressive stress values was justified. However, there was a limitation due to the lack of experimental results. Based on the results presented in the previous subsection and in Figure 9, it can be seen that the Papadopoulos criterion guaranteed a small and acceptable error over the entire range of the experiment. For this reason, it was decided to estimate the critical values of alternating shear stress amplitude $\tau_{a}$ using the Papadopoulos criterion for the given compressive stress values. The loading conditions were assumed to be the same as in Section 3.2—$\sigma_{a}=\;\left|\sigma_{m}\right|$ and a shift in phase between the compressive and the shear stresses equal to 90°. These calculations were performed for $\sigma_{a}=\;-\sigma_{m}$ in the range of 0–536 MPa with a step of 50 MPa, and for steel R7T with properties $a_{DV}=0.3$ ($t_{-1}=310\;MPa;\;f_{b-1}=517\;MPa$). The obtained results were supplemented with the results from the experiment in [22] and are reported in Figure 10.

The absence of a bar in the graph should be interpreted as meaning that, in a given case, the error is 0%. This situation occurred for point no. 1, which is the classical alternating fatigue torsion test, and in this case, the error value should be exactly 0. This is due to the fact that this fatigue test was used for the calibration of the MFC. In the case of the Papadopoulos criterion, relative errors were reported only for points no. 2 and no. 3 because only in these cases were the calculations performed based on the experimental data. In the case of points 4–9, the loading conditions were determined using the Papadopoulos criterion, so the error values were obligatorily equal to 0%. Here, the values of the permissible shear stress amplitude $\tau_{a}$ were calculated for the assumed values of the pulsating compression to obtain a relative error δ equal to 0 (Equation (20)).

In the whole investigated range, the original DV criterion was the most non-conservative one. The application of this model led to the highest underestimation of the fatigue effort with reference to the experimental tests and the Papadopoulos criterion at each investigated point. The greatest errors were obtained when the ratio ξ of the maximal compressive stress and shear stress amplitude, $\xi =\sigma_{max\_c}/\tau_{a}=\left(\sigma_{a}+\left|\sigma_{m}\right|\right)/\tau_{a},$ was in the range between three and four (it corresponds to points 8 and 9). The mean relative errors for all loading conditions shown in Figure 10 are listed in Table 5.

### 3.4. Multiaxial Fatigue Analysis of Thrust Roller Bearing

**Figure 11:**
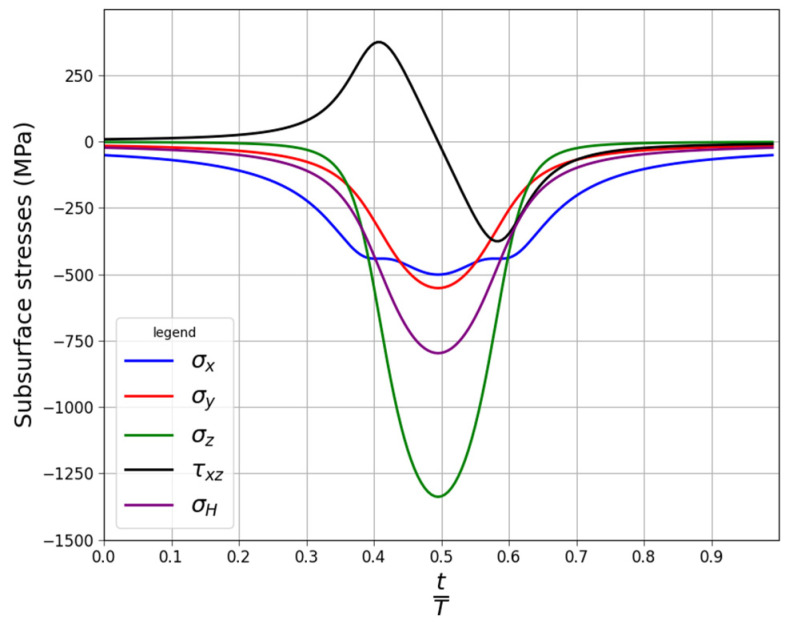
Subsurface stresses in roller on critical radius (one stress cycle): bearing, K81102 TN; loading, 2.45 kN.

An analysis was performed for the cylindrical roller thrust bearing, with the designation K 81102 TN [50]. The principal dimensions of the bearings were as follows: the bore diameter,15 mm; the outside diameter, 28 mm; the height, 3.5 mm; and the basic dynamic load rating, 11.2 kN. According to the data provided by the manufacturer, the bearing’s fatigue load limit was equal to 2.45 kN. The bearing consisted of 12 rolling elements, which were rollers with a radius of 1.75 mm. Based on the study [37], it was assumed that the flat length of the roller (where there was contact between the roller and the rings) was equal to 80% of the roller length, which corresponded to 1.9 mm. The subsurface contact stresses were calculated with the use of the solution proposed by Radzimovsky [27,37,51]. The validation of the analytical solution by means of the finite element method was performed in Ref. [27]. The stress distributions were calculated at the critical radius for 200 points. The distribution of the subsurface stresses for one stress cycle at the critical radius is shown in Figure 11. The critical radius should be understood as the radius at which the stresses leading to the highest fatigue stresses occur. The location of the critical radius was evaluated with the use of the Papadopoulos criterion [14].

The fatigue analyses were made assuming that the bearing was made of AISI 52,100 bearing steel (the main alloying components, in weight%: C—0.95–1.05; Cr—1.30–1.65; Si—0.15–0.35; and Mn—0.25–0.45). The mechanical properties of the steel were as follows: the ultimate tensile strength, 2250 MPa; the Yield limit, 2000 MPa; the Young modulus, 210 GPa; and Poisson’s coefficient, 0.3. The fatigue properties of the material were set with respect to the recommendations presented in Ref. [37]. The calculations were performed assuming that the bearing was subjected to a catalogue fatigue load limit (2.45 kN). Due to the small diameter of the roller, the size factor $k_{s}=1$. The size factor was also included in the DV criterion according to the relationship

(22) $$\tau_{DV}\left(t\right)=\frac{1}{k_{s}}\tau \left(t\right)+a_{DV}\cdot \sigma_{H}\left(t\right)\leq t_{-1}.$$

The results were presented in the form of a safety factor $x_{s}$, in which the fatigue stress $\tau_{fat}$ calculated using a particular criterion was related to the admissible alternate torsion fatigue strength $t_{-1}$ of the material. Here, three cases can be distinguished:

- The criterion underestimates the fatigue effort—$x_{s}<1$;
- The criterion provides a result consistent with the catalogue data—$x_{s}\approx 1$;
- The criterion overestimates the fatigue effort—$x_{s}>1$.

Generally, the properties of bearings (e.g., basic dynamic load rating) are given for a rating life of one million rolling bearing revolutions with 90% reliability. The fatigue material properties ($t_{-1}$ and $f_{-1}$) for a certain number of cycles (corresponding to one million revolutions) of AISI 52100 bearing steel were calculated using the Wöhler curves, assuming the same conditions. The number of cycles were calculated using the geometry of the bearing and based on the determined number of cycles. The fatigue limits for alternate bending and torsion were calculated from the S-N diagrams using the formulas given below [37,52]:

(23) $$\begin{array}{c} t_{-1}=2580N_{f}^{-0.103}\\ f_{-1}=2200N_{f}^{-0.0594} \end{array}$$

The above S-N curves were determined based on the research conducted by Shimizu et al. [53] and Saki [54]. The Papadopoulos criterion requires the integration of stresses in all possible orientations of the material plane, and the DV criterion requires the determination of the critical plane. In both cases, the calculations were performed in 5-degree increments. The obtained results are reported in Table 6.

## 4. Discussion

The original DV criterion has often been criticised for overestimating the effect of compressive hydrostatic stresses on the fatigue effort. This problem was also confirmed in this study. In each of the analysed cases, the original DV criterion gave the largest relative errors (underestimation of the fatigue stress) due to the overestimation of the influence of the compressive stresses. These errors reached values of up to −35% for alternating torsion with pulsating compression loading (see Figure 7 and points no. 8 and 9 in Figure 10) and −40.3% in the case of roller bearing. Moreover, it was also noticed that this overestimation strongly depended on the fatigue material properties used for the determination of $a_{DV}$ coefficient (see Table 4). The use of the fatigue limit for tension–compression ($f_{tc-1}$) to analyse problems with high compression stresses leads to significant calculation errors. Because of this, it is a non-conservative approach, which reduces the level of security. The use of the fatigue limit for alternating bending ($f_{b-1}$) reduced this error (up to −9.38% compared to −15.83% for the case in which $f_{tc-1}$ was used); however, the fatigue stress was still underestimated.

The example using the DV mod. 3 shows that the omission of the compressive hydrostatic stress, despite some improvement, does not solve the problem of underestimating the fatigue effort when a compressive stress exists. In each investigated case of the experimental tests (Figure 7, Figure 8 and Figure 9), this error increased with an increasing compressive stress, similarly to the original version of the DV criterion. This trend was also observed for higher compressive stresses (see loading conditions no. 6–9 in Figure 10). For a certain level of pulsating compression (point 10 in Figure 10), the obtained results were the same as for the original DV criterion. This modification is insensitive to the calibration method (the coefficient $a_{DV}$ has no effect on the calculations) when compression stresses occur (see Table 4). The use of this model for RCF analysis of roller bearings resulted in an underestimation of the fatigue effort by about −22.2%.

Desimone’s proposal (DV mod. 2), in which the compressive stress is neglected and the fatigue limit is reduced from $t_{-1}$ to $0.5f_{b-1}$, guarantees a conservative estimation of low-to-medium compressive stresses. This can be seen in the comparison with the experiments—Table 4 and loading conditions no. 1–7 in Figure 10. However, the fatigue effort is significantly overestimated. In the case of high compressive stresses (loading conditions no. 8–10 in Figure 10), this variant also underestimates the fatigue effort compared with the Papadopoulos criterion. However, the underestimation error is significantly smaller than for the original DV and the DV mod. 3 (up to −20%). This effect has an important meaning for RCF analyses. This problem is illustrated by the example of roller bearing, in which the fatigue stress is still underestimated by about −11.2% (Table 6). On the other hand, the model ensures high accuracy when the pulsating compression reaches extremal admissible values (loading condition no. 11—12 in Figure 10). Similar to the DV mod. 3, this modification is also insensitive to the calibration method when compression stresses occur (see Table 4). Another disadvantage of this approach is that the DV mod. 2 does not meet the conditions for alternating torsion.

The best agreement with the experiments (the absolute error 0.77%—Table 4) as well as with the Papadopoulos criterion (the absolute error 5.8%—Table 5) is obtained for the DV mod. 4, with $\;a_{c}=1$. Here, it is assumed that the compressive stress has the same negative influence on the fatigue strength as the tensile stress. In consequence, unlike the other investigated versions of the DV criterion, the compressive hydrostatic stress increases the fatigue effort by adding to the shear stress. Comparing the results obtained by this version of DV mod. 4 ($a_{c}=1)$ with those for cases with $a_{DV}=0.996$ (Figure 7) and $a_{DV}=0.3$ (Figure 8), it can be seen that the use of $f_{tc-1}$ leads to more conservative results than for the case in which $f_{b-1}$ is used. This difference is directly related to the value of the $a_{DV}$ coefficient, which takes into account the influence of hydrostatic effects. When anisotropy is not included and $f_{tc-1}$ is used for the calculation of $a_{DV}$, the smallest error is obtained for $a_{c}=0.5$—Table 4. In comparison with the Papadopoulos criterion, it can be seen that a slight underestimation occurs for high pulsating compression (points 6–9 in Figure 10). This modification overestimates the fatigue stress for extremal pulsating compression (points 11 and 12 in Figure 10). Both DV mod. 4 (with $a_{c}=1)$ and the Papadopoulos criteria give similar fatigue stresses for the investigated roller bearing (Table 6). This result is also consistent with the catalogue data. The relative error for the DV mod. 4 with $a_{c}=1$ is equal to 1.1%. For smaller values of $a_{c}$ (0.8 and 0.5), the fatigue stress is underestimated by 3.8% and 11.3%, respectively, compared to the catalogue data.

Summarising, the application of an appropriate modification of the DV criterion with the simultaneous use of the proper material data for calibration allows for accurate results of fatigue analyses for RCF issues to be obtained. For the experiment investigated in this study, the highest accuracy for problems with non-proportional loadings with compressive stresses was achieved for the DV mod. 4 with $a_{c}=1$.

## 5. Conclusions

The original DV criterion based on the critical plane approach and its different modifications are investigated in this study in the context of applications to RCF issues. Validation of all variants is performed with the use of experimental results (alternating torsion with out-of-phase pulsating compression), data calculated by means of Papadopoulos criterion and the example of roller bearings. On this basis, the following conclusions can be drawn:

(1) The original DV criterion, based on the critical plane approach, underestimates the fatigue stress when compressive stress occurs. Higher compressive stresses lead to a higher underestimation of fatigue stress. For this reason, this criterion is not suitable for RCF analyses.
(2) The DV criteria (except the DV mod. 2 and the DV mod. 3 in the case of analyses of problems in which compressive stress occurs) are vulnerable to the material properties used during the calibration. An improper calibration may result in large calculation errors. For RCF analyses, it is recommended to use the alternative fatigue bending limit $f_{b-1}$.
(3) Neglecting the influence of compressive stresses (the DV mod. 3) does not solve the problem of underestimation of the fatigue stress for RCF issues.
(4) The DV mod. 2 (with the critical plane approach) is too conservative for loadings with small compression effects and underestimates the fatigue stress for high pulsating compression. Despite significantly smaller errors than the original DV and DV mod. 3 criteria, the use of this variant for RCF analyses may also result in underestimation of the fatigue stress.
(5) The highest agreement with the experimental results, the Papadopoulos criterion and, in the case of the practical application (roller bearing), is obtained for the proposed modification DV mod. 4 (with $a_{c}=1$), in which compressive hydrostatic stress is assumed as unfavourable for the fatigue process.
(6) Due to the narrow validation of the criteria, in the case of its application to other materials, it is recommended to validate the proposed modification using out-of-phase fatigue loading experiments with three-dimensional compression and alternating torsion.

It should be noted that the proposed modifications are only verified for specific stress conditions (out-of-phase loading, three-dimensional compression with alternating shear stresses) that occur in the components subjected to rolling contact. The $a_{c}=1$ assumption, though physically motivated and empirically supported within the available dataset, requires validation across a broader range of materials and loading conditions. For other applications, further verification of the presented models is necessary.

## Figures and Tables

**Table 1:** Influence of material properties on Dang Van $a_{DV}$ coefficient.

Designation	$f_{tc-1}$ (MPa)	$f_{b-1}$ (MPa)	$t_{-1}$ (MPa)	$a_{DV(t-c)}$	$a_{DV(b)}$
S235JR + N [49]	140	180	105	0.750	0.250
S275JR + N [49]	170	215	125	0.706	0.244
S355JR + N [49]	205	255	150	0.695	0.265
E295 [49]	195	245	145	0.731	0.276
E335 [49]	235	290	180	0.798	0.362
E360 [49]	275	345	205	0.736	0.283
C22E + QT [49]	200	250	150	0.750	0.300
C40E + QT [49]	260	325	200	0.808	0.346
34Cr4 + QT [49]	360	450	270	0.750	0.300
34CrMo4 + QT [49]	400	500	300	0.750	0.300
30CrNiMo8 + QT [49]	500	625	375	0.750	0.300
R7T [22]	375	520 ^(1)^	312	0.996	0.300
41Cr4 [49]	400	500	300	0.750	0.300

^(1)^: Value evaluated using formula $t_{-1}=0.6f_{b-1}$; it corresponds to $\frac{f_{tc-1}}{f_{b-1}}=0.72$.

**Table 2:** Influence of $a_{DV}$ coefficient on fatigue results obtained by the use of original DV criterion: 30CrNiMo8 + QT steel, out-of-phase loading conditions.

No.	Applied Loading			Equivalent Fatigue Effort $\tau_{DV}^{MAX}\;(MPa)$	
	$\sigma_{a}\;(MPa)$	$\sigma_{m}\;(MPa)$	$\tau_{a}\;(MPa)$	$a_{DV(t-c)}=0.75$	$a_{DV(b)}=0.30$
1	0	0	300	300	300
2	200	200	300	354	321
3	400	400	300	428	345
4	200	−200	300	254	281
5	400	−400	300	228	265
6	500	−500	300	250	263
7	600	−600	300	300	300
8	700	−700	300	350	350

**Table 3:** Fatigue limits of R7T wheel steel used in validation of Dang Van criteria.

No.	Amplitude of Compressive Stress $\;\sigma_{a}\;(MPa)$	Mean Value of Compressive Stress $\sigma_{m}\;(MPa)$	Amplitude of Shear Stress $\tau_{a}\;(MPa)$
1	0	0	297
2	100	−100	287
3	200	−200	267
4	0	0	310
5	100	−100	301
6	200	−200	293

**Table 4:** Mean relative errors for tests No. 1–No. 6.

No.	Mean Relative Errors $\delta_{mean}$$\;\mathrm{and}\;\delta_{m\_abs}$ (%) for Tests No. 1–No. 6					
	Anisotropy Not Included				Anisotropy Included $a_{DV}=0.3,$ $f_{b-1}$	
	$a_{DV}=0.996,$ $f_{tc-1}=375\;(MPa)$		$a_{DV}=0.3,$ $f_{b-1}=520\;(MPa)$			
	$\delta_{mean}(\%)$	$\delta_{m\_abs}(\%)$	$\delta_{mean}(\%)$	$\delta_{m\_abs}(\%)$	$\delta_{mean}(\%)$	$\delta_{m_{abs}}(\%)$
Papadopoulos	−3.21	3.52	−3.21	3.52	−0.52	1.04
Original DV	−15.83	15.83	−9.38	9.38	−6.85	6.85
DV mod. 2	12.50	12.50	12.50	12.50	15.59	15.59
DV mod. 3	−6.25	6.25	−6.25	6.25	−3.64	3.64
DV mod. 4 ${(a}_{c}=1)$	5.46	7.28	−2.97	3.15	−0.27	0.77
DV mod. 4 ${(a}_{c}=0.8)$	2.91	4.73	−3.64	3.64	−0.96	0.96
DV mod. 4 ${(a}_{c}=0.5)$	−0.67	3.03	−4.64	4.64	−11.94	11.94

**Table 5:** Mean relative errors for tests presented in Figure 10.

No.	$\delta_{mean}(\%)$	$\delta_{m\_abs}\;(\%)$
Original DV	−16.9	16.9
DV mod. 2	4.0	11.7
DV mod. 3	−13.3	13.3
DV mod. 4 ${(a}_{c}=1)$	0.7	5.8
DV mod. 4 ${(a}_{c}=0.8)$	−2.2	5.8
DV mod. 4 ${(a}_{c}=0.5)$	−6.5	7.2

**Table 6:** Mean relative errors of RCF calculations for K 81102 TN bearing ($t_{-1}=481\;MPa$; $f_{b-1}=843\;MPa$).

No.	$x_{s}=\tau_{fat}/t_{-1}$	δ
Papadopoulos	1.0	0%
Original DV	0.60	−40.3%
DV mod. 2 ^(1)^	0.89	−11.2%
DV mod. 3	0.78	−22.2%
DV mod. 4 $(a_{c}=1$)	1.01	1.1%
DV mod. 4 $(a_{c}=0.8$)	0.96	−3.8%
DV mod. 4 $(a_{c}=0.5$)	0.89	−11.3%

^(1)^: In this criterion, the fatigue limit is equal to $0.5f_{b-1}=421.5\;MPa$.

## Data Availability

The original contributions presented in this study are included in the article. Further inquiries can be directed to the corresponding author.

## Abbreviations

The following abbreviations are used in this manuscript:

DV	Dang Van
RCF	Rolling contact fatigue
MFC	Multiaxial fatigue criteria
